# Jaccoud's Arthropathy in a Case of Mixed Connective Tissue Disorder: An Interesting Tale

**DOI:** 10.7759/cureus.33170

**Published:** 2022-12-31

**Authors:** Jahnabi Bhagawati, Mansi Patel, Keyur Saboo, Sunil Kumar, Sourya Acharya

**Affiliations:** 1 Department of Medicine, Jawaharlal Nehru Medical College, Datta Meghe Institute of Medical Sciences, Wardha, IND; 2 Department of Internal Medicine, Jawaharlal Nehru Medical College, Datta Meghe Institute of Medical Sciences, Wardha, IND

**Keywords:** rheumatoid like, hand deformities, interstitial lung disease, mixed connective tissue disease, jaccoud’s arthropathy

## Abstract

In Jaccoud's arthropathy (JA), classical deformities that resemble rheumatoid arthritis (RA), such as swan neck deformity and ulnar deviation, are present, but in contrast to RA, these are *reducible* to passive movement and nonerosive arthritis. Similar deformities are also described in various connective tissue disorders. Mixed connective tissue disease (MCTD) characteristically has overlapping features of various connective tissue diseases (CTDs) such as systemic lupus erythematosus, systemic sclerosis, and myosotis with an association of U1RNP antibody. Approximately 60% of patients with MCTD may have deforming arthritis. We present a case of a 23-year-old male, with multiple overlapping clinical features and deforming arthritis. This case begs the question to identify various possible differentials of deforming arthritis.

## Introduction

In 1869, Francois-Sigismond Jaccoud, a physician, described a young patient with rheumatic fever (RF) and chronic joint deformities. Hence, as a tribute to this physician, these deformities are known as Jaccoud's arthropathy (JA). Classical deformities that are associated with it are swan neck deformity, ulnar deviation, thumb subluxation, and *boutonniere* deformity, similar to those seen in rheumatoid arthritis (RA) but are different as these deformities are characteristically *reducible* to passive movement. In addition, there are no joint erosions in a plain radiograph. Nowadays, RF is rarely seen. JA has been associated with several disorders, particularly systemic lupus erythematosus (SLE). JA has also been described in connective tissue disorders such as systemic sclerosis (SSc), polymyositis, Sjogren's syndrome, and vasculitis as well as in normal individuals [1].

In 1972, Sharp et al. initially described mixed connective tissue disease (MCTD) as a syndrome, which was unique in its overlapping features of SLE, SSc, and myositis with an association of U1RNP antibody [2]. In Norway, a study was conducted to estimate the incidence and prevalence of MCTD, which was found to be 2.1 per million per year and 3.8 per 100,000 adults, respectively, in the Norwegian population [3]. In the study, the female-to-male ratio was found to be 3.3, and the mean age at diagnosis was 37.9 years. MCTD can be associated with a myriad of clinical manifestations and may present in an elusive way. These manifestations may not be the presenting feature all at once, and patients may recruit different clinical features with different timelines. MCTD has been found to have high titers of U1RNP antibodies along with manifestations like Raynaud’s phenomenon, pulmonary hypertension, renal involvement, arthritis, interstitial lung disease, and myositis. Data collected worldwide has been found to have an incidence of deforming arthritis of approximately 60% with MCTD [4]. Patients either presenting with Raynaud's phenomenon, arthritis, myositis, ILD, sclerodactyly, fever, and/or fatigue or developing any combination of these symptoms throughout their disease, not fitting into criteria for any particular connective tissue disease with high titers of U1RNP, should alert the physician to consider MCTD in the list of differentials.

## Case presentation

A 23-year-old male patient came to the clinic with complaints of generalized fatigue for four years. The patient also complains of pain and swelling of the joints of both hands for the past two years, which was not associated with morning stiffness. There is a history of dry cough on and off for the past year. The patient also has had difficulty opening his mouth for one year. There is no history of chest pain, palpitation, recurrent oral ulcers, hair loss, fever, rash, or weakness in the limbs. On further history taking, the patient gave a history of bluish discoloration of fingers on exposure to cold water.

The patient was admitted due to chronic dry cough and administration of injectable antibiotics.

**Figure 1:**
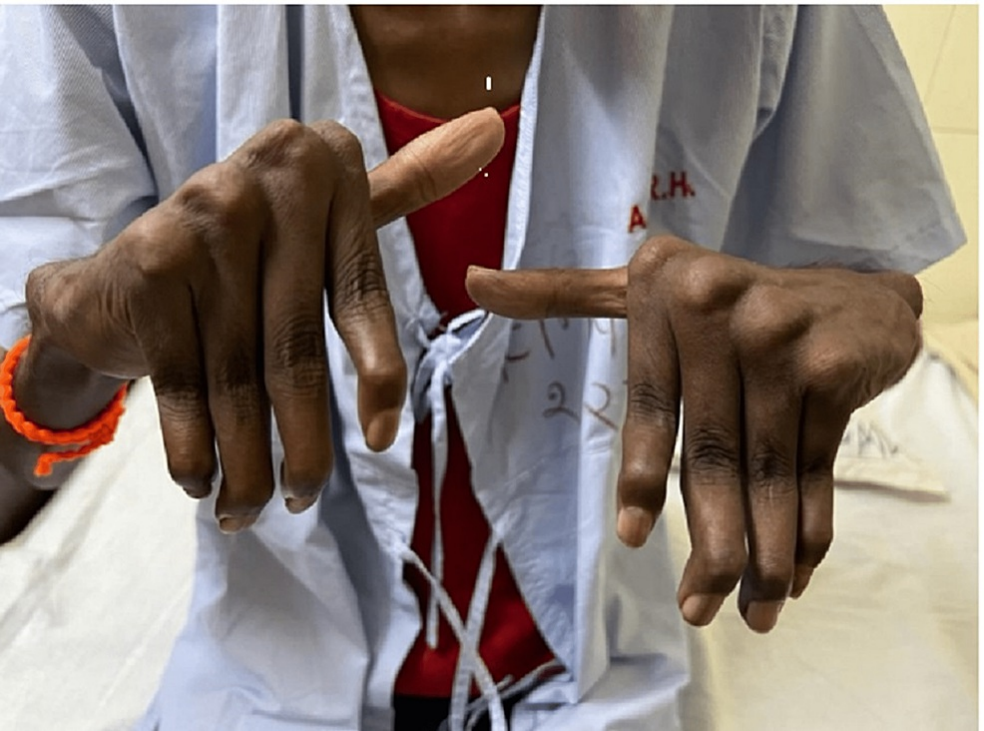
Jaccoud's arthropathy.

On examination, the patient was thin-built, with deformities in both hands, which had flexion deformities and ulnar deviation at the metacarpophalangeal joints with hyperextension at proximal interphalangeal joints. The deformity (unlike RA) was not a fixed joint deformity, which was nontender (Figure 1). The patient was able to do his routine activity with his hands with some difficulty, but on passive movements, the deformity was partially reducible. The patient was oriented to time, place, and person. The pulse rate was 90 beats per minute, and the blood pressure was 110/70 mmHg in the right arm supine position, with a respiratory rate of 22 cycles per minute. On respiratory system examination, coarse leathery crepitations were present in the bilateral lung. His cardiovascular examination revealed normal first and second heart sounds and no murmur. The abdomen was normal on examination. During the central nervous system examination, the patient was conscious and oriented.

Based on the aforementioned history, the patient was suspected of having a connective tissue disorder for which he was investigated. All the investigation profiles are given in Table 1.

 

**Table 1:** Investigation profile of the patient.

Investigation	Patient’s report	Reference values
Hemoglobin	10.9 g/dL	13-17 g/dL
Erythrocyte sediment rate	40	0-20
Peripheral smear	Normocytic normochromic	Normocytic normochromic
Serum creatinine	1.1 mg/dL	0.6-1.25 mg/dL
Albumin	2.4 gm/dL	3.5-5.0 g/dL
Aspartate aminotransferase	12 units/L	<50 units/L
Alanine aminotransferase	30 units/L	17-59 units/L
Total bilirubin	0.3 mg/dL	0.2-1.3 mg/dL
Autoantibodies		
Anti-nucleoside antibody	Positive	-
Anti-cyclic citrullinated peptide	Weakly positive	-
Rheumatoid factor	Weakly positive (70 units/mL)	-
Anti-U1 ribonucleoprotein antibody	Positive	-
Anti-double-stranded deoxyribonucleic acid antibody	Negative	-
Anti-Smith antibody	Negative	-
Anti-topoisomerase 1 antibody	Negative	-

**Figure 2:**
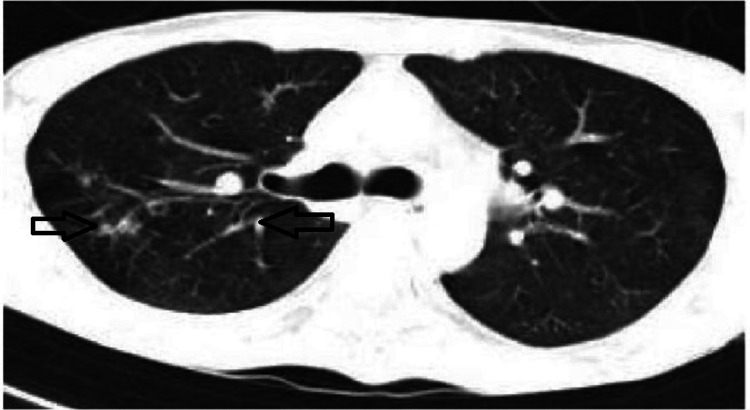
HRCT thorax revealed ground glass opacity. HRCT, high-resolution computed tomography

Because of chronic cough, high-resolution computed tomography of the thorax was conducted, which revealed ground glass opacity, suggestive of interstitial lung disease (Figure 2).

**Figure 3:**
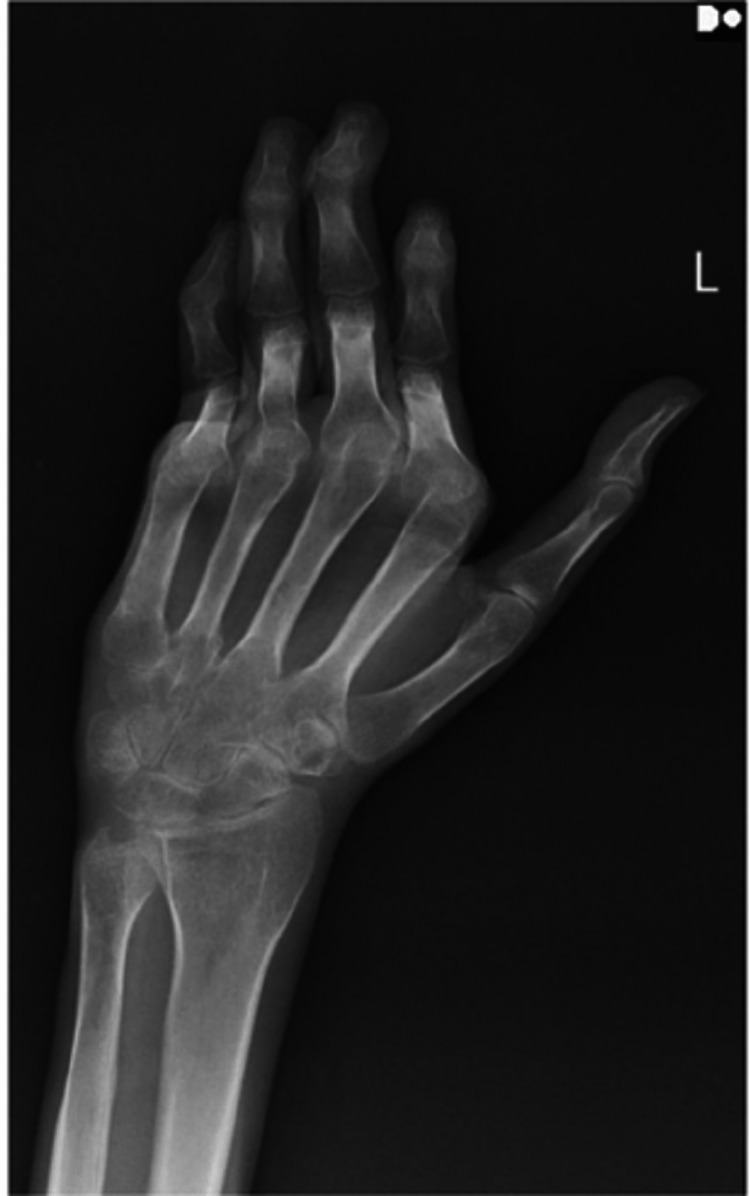
X-ray revealing nonerosive deformity.

His pulmonary function test was suggestive of restrictive lung disease as the forced expiratory volume in 1 second (FEV1)/forced vital capacity (FVC) ratio was 85%, FVC was 2.5 L, and total leukocyte count (TLC) was <75%. Two-dimensional (2D) echocardiography had diastolic left ventricular dysfunction and normal systolic function. Also, the right ventricular systolic function was 19 mmHg. His hand X-ray showed nonerosive deformity of joints (Figure 3).

**Figure 4:**
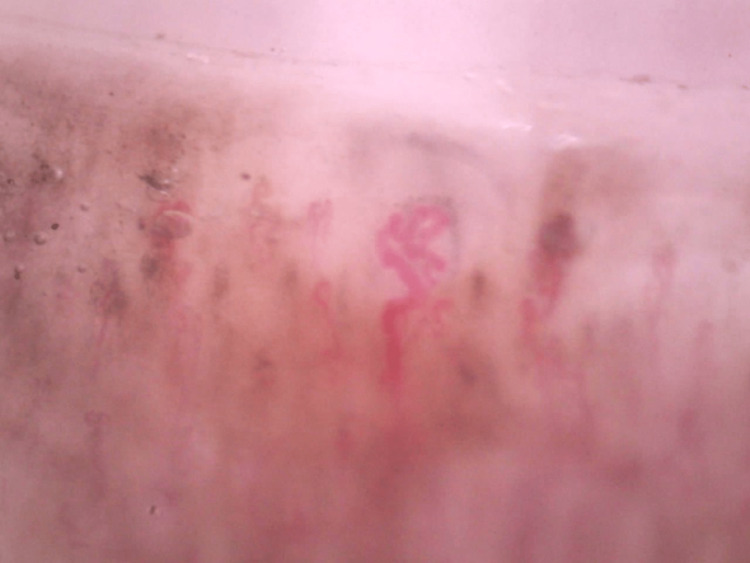
Nailfold capillaroscopy showing dilated capillaries.

A nailfold capillaroscopy was done, which revealed dilated capillaries (Figure 4).

Hence, the patient was diagnosed to have mixed connective tissue disorder (based on antinuclear antibody, or ANA; anticyclic citrullinated peptide, or CCP; RA factor; anti-U1RNP-positive; and nailfold capillaroscopy, as shown in Figure 4, with JA).

The patient was started on tab prednisolone 60 mg once a day, tab methotrexate 20 mg per week, folic acid 5 mg three times a week, and vitamin 60 IU per week. The patient was asked to come for follow-up, and after one month, he was doing well in terms of reduced cough and well-being; deformities were present but compatible with lifestyle modifications; and he was doing daily activities by both hands with slight difficulty. The patient did not have pain and any other joint-related problems.

## Discussion

JA is a form of arthritis that can lead to reducible deformities of the hands and feet (less frequently knees, shoulders, and wrists), which are different from those in RA [5].

The deformities classically seen in JA, such as thumb subluxation, swan neck, ulnar deviation, and *boutonniere*, are similar to those observed in RA but are characterized by reducibility to passive movement as well as no joint erosions on plain radiographs. JA has been found to have an association more commonly with SLE but also with connective tissue disorders, such as SSc, polymyositis, Sjogren's syndrome, vasculitis, and neoplastic diseases, and less frequently even in normal individuals.

Although a *reducible *pattern of arthropathy is observed in the majority of JA cases, in most advanced cases, the joints may become fixed, sometimes referred as to *severe JA*, which is clinically difficult to distinguish from RA [1].

CTD, a clinical entity belonging to a heterogeneous group of immunologically mediated disorders, is characterized by inflammation, tissue damage, and abnormal repair, leading to the dysfunction of the target organ, replacement by fibrotic tissue, and loss of organ functioning. The origin of these disorders is multifactorial, having a complex interplay between genetic and environmental factors [6].

In 1971, Sharp et al. recognized a clinical disorder that they named MCTD [7]. They have clinical manifestations typical of SLE, SSc, and polymyositis but without meeting clear diagnostic criteria for those diseases. The occurrence of anti-ribonucleoprotein (RNP) antibodies in the patient's serum is considered an immunological marker of MCTD. The disease is caused by several factors such as genetic factors (presence of the human leukocyte antigen, or HLA, DRB1 and histocompatibility antigens HLA-DR4 and HLA-B8), environmental factors (cytomegalovirus, Epstein-Barr virus, retroviruses, exposure to chemical factors such as vinyl chloride, female sex hormones, and some drugs, e.g., procainamide), and congenital and inborn immune response disorders [7].

Patients may manifest symptoms of more than one disease simultaneously or may develop manifestations of different diseases sequentially. Although for the classification of MCTD there are no American College of Rheumatology criteria, a few clinicians favor the criteria proposed by Alarcon-Segovia and Cardiel [8]. Serologically, a very high titer of ANA and anti-U1-RNP can give the first hint to the diagnosis. The absence of other specific autoantibodies is important too. Antibodies to anti-Smith antibodies, double-stranded DNA (dsDNA), and anti-Ro/anti-La are sometimes seen as transient phenomena [9]. Patients may present with nonspecific symptoms early in the course of diseases, such as fatigue, malaise, myalgias, arthralgias, and Raynaud's phenomenon. Many of the organ systems may be involved in MCTD during illness. More than 90% of the patients were observed to have arthralgias, and 50% to 60% had arthritis. Rheumatoid factor and anti-CCP antibodies may be positive. Hematologic abnormalities in these patients seen are anemia, leukopenia, lymphopenia, and thrombocytopenia. Pulmonary symptoms such as cough, dyspnea on exertion, or pleuritic chest pain can occur in 75% of these patients, with the chances of a physical examination demonstrating basilar rales. Interstitial lung disease and cryptogenic pneumonitis had been found in up to 50% of these patients [10].

## Conclusions

MCTD lies in a gray zone among disorders such as RA, SLE, SSc, and myositis. Although MCTD can present with symptoms typical of a disease such as lupus, scleroderma, and RA, it may not fit into one particular disease as per their criteria. Deforming arthritis being one of these features must be critically examined for reducibility and radiographic erosions as they may mimic RA. Hence, time itself may reveal the true nature of the disease when patients show overlapping features of several CTDs with a strong association of U1-RNP, ultimately falling into the domain of MCTD.
